# The Perspectives of Professional Caregivers on Implementing Audio-Based Technology in Residential Dementia Care

**DOI:** 10.3390/ijerph17176333

**Published:** 2020-08-31

**Authors:** Maarten Houben, Rens Brankaert, Gail Kenning, Berry Eggen, Inge Bongers

**Affiliations:** 1 Department of Industrial Design, Eindhoven University of Technology, 5600 MB Eindhoven, The Netherlands; r.g.a.brankaert@tue.nl (R.B.); j.h.eggen@tue.nl (B.E.); 2 Tranzo, School of Social and Behavioral Sciences, Tilburg University, 5000 LE Tilburg, The Netherlands; i.m.b.bongers@tilburguniversity.edu; 3 School for Allied Health Professions, Fontys University of Applied Sciences, 5600 AH Eindhoven, The Netherlands; 4 Ageing Futures Institute, University of New South Wales, Sydney, NSW 2052, Australia; gail.kenning@unsw.edu.au; 5 Mental Healthcare Institute Eindhoven, 5626 ND Eindhoven, The Netherlands

**Keywords:** audio-based technology, care practice, dementia, participatory workshops, professional caregivers, sound, technology in healthcare

## Abstract

Music and familiar everyday sounds can be meaningful for people with dementia by providing benefits such as evoking memories and emotions or prompting social interactions with caregivers or relatives. Motivated by this potential, researchers and designers are investigating how to leverage these beneficial effects of sound in care environments through audio-based technology. However, there is a gap in the knowledge of how audio-based technology can be successfully implemented within everyday care practice. In this paper, we present the outcome of three participatory workshops with 18 professional caregivers to explore how audio-based technology can add value to existing care processes and activities in residential dementia care. During the participatory workshops, professional caregivers (1) mapped existing care activities; (2) linked findings in research with practice, and (3) designed scenarios for the Vita sound cushion. Care professionals indicate how audio-based technology can support existing care practice by influencing the mood of residents and by supporting social interaction during moments of care, daytime activities, or situational sessions. This study bridges research findings with insights from practice, contributing to a shared understanding of opportunities for embedding audio-based technology in dementia care. These opportunities motivate future research to implement and evaluate audio-based technology in residential dementia care.

## 1. Introduction

Dementia is a societal health challenge, as the global population is aging and the number of incidences rises [1]. Without a cure and with a lack of effective pharmacological interventions [2], there is an increasing need for nonpharmacological alternatives to increase the quality of the everyday lives of people with dementia [3]. Over the years, researchers from various disciplines have demonstrated the beneficial effects of music for people with dementia [4,5,6,7]. For instance, music can reduce stress and agitated behavior [8] and can positively influence residents’ emotional states and mood in residential dementia care [5,9]. Furthermore, music from a person’s past can evoke autobiographical memories [10,11,12] and can support recognizing and maintaining identity [13,14]. Alongside listening to music, people with dementia can also participate actively in music sessions by playing instruments, by singing, or by moving to music [6]. Therefore, music sessions can stimulate physical activity [15,16] and can provide opportunities for social interaction with professional caregivers and other residents in a care home [7,17,18].

Building on this extensive body of work on music in dementia [4,5,6,7,8,9,10,11,12,13,15,16,17,18], researchers have begun exploring the potential benefits of everyday sounds or nonmusical sounds perceived in everyday life for people with dementia [19,20,21,22,23,24,25]. Like music, everyday sounds that are perceived as pleasant can evoke positive emotions [24,26]. For instance, sounds of nature can relax and calm people with dementia [25]. Reexperiencing sounds from everyday life can provide feelings of comfort or safety [27], can prompt memories of life events and loved ones [20], and can support storytelling by people with dementia to relive meaningful past experiences in the present [18,19]. These responses to sound provide opportunities for social connection and interaction [21,22] and for improvement in the living environment of care facilities, where sounds can be perceived and enjoyed by residents regardless of their limitations or illness [23].

Motivated by the potential of music and everyday sounds in dementia, researchers from various disciplines investigated how to leverage these beneficial effects of sound into everyday care by using audio-based technology (ABT). Soundscapes or perceived collections of ambient sounds can augment the existing sonic environment with pleasant and familiar sounds to provide a sense of safety and structure for residents in residential dementia care [28,29,30]. For example, introducing soundscapes that contain nature sounds, such as bird songs or rustling tree leaves, to care home residents in the morning can cue time-related activities, such as waking up and getting dressed [31]. Moreover, researchers have been exploring the role of sound in daytime activities to stimulate social engagement and creativity [32], such as art therapy [33] and music sessions [6]. These research explorations have resulted in new accessible interfaces for people with dementia to enable them to play personal music content [34,35,36] and virtual instruments adapted to the skills and abilities of residents with dementia to support participation during music therapy sessions [7,17,37,38]. ABT, such as music pillows [22,29] and collaborative interfaces [17,21,39], support social activities and stimulate engagement between people with dementia, their relatives, and professional caregivers [21,22].

Initial research that explored the perspectives of professional caregivers on sound in care practice has indicated that ABT can alleviate part of their workload and can improve the quality of life of people with dementia by decreasing stress and agitation for people with cognitive impairments in general by providing a sense of safety, daily structure, and comfort [40]. However, there is a lack of understanding on how these interventions can be purposefully used in existing care processes. Professional caregivers suggest that the implementation of interventions involving sound in residential dementia care should not result in unintended behavior of residents, such as unrest [28]. For example, sound is not always desirable during moments in care that require silence, such as resting activities, or moments when ambient or incidental sounds are already noticeable, for example, when breakfast is served [28]. Overall, professional caregivers struggle to successfully incorporate novel technologies such as ABT into dementia care practices [41]. Research shows that technologies for health care settings in general often seem successful and show promise at first yet lack sustainability, and repeatedly fail to become implemented into health care programs [41,42]. Time constraints and heavy workloads are recurring barriers for the care staff in accommodating new technologies into their current care routines [42]. Therefore, while there is an urgent need for integrated care services that address the needs of both people with dementia and their caregivers, integrating technology into dementia care remains a complex process because of the various stakeholders and contextual factors [41]. More insights are needed into the lived experiences and everyday needs of working in residential dementia care to investigate how professional caregivers can successfully embrace ABT.

By adopting a participatory approach, we aim to contribute to existing research by mapping the perspectives of professional caregivers on opportunities for ABT to augment existing activities, routines, and habits in residential dementia care with meaningful audio content. A participatory design is established in the literature as an effective method to gain insight into people’s values, beliefs, preferences, and everyday living or working environments [43]. Participatory approaches have been applied in the context of health care interventions to assess the attitudes and perspectives of professional caregivers on the acceptance of new technologies or organizational innovations [44,45,46]. For instance, to gain a qualitative understanding of the everyday working experience within healthcare teams, such as care tasks, different or common goals, and interdependencies with other teams [45].

In this paper, we discuss and present the outcomes of three participatory workshops where 18 professional caregivers divided over three sessions explored and mapped opportunities of ABT in residential dementia care. With our workshop approach, we aimed to bridge insights from relevant academic literature with personal lived experiences and expertise of care professionals. The participatory workshop approach focused on the professional caregivers’ perspectives in using ABT to enrich and contextualize our previous research efforts on in-context responses of people with dementia on everyday sounds in dementia [19,22]. The results of our field study describing the in-context responses of the residents with dementia have been reported and published in other literature [22]. In this paper, we contribute the outcomes of the participatory workshops that preceded the field study and discuss the opportunities for ABT in dementia care that emerged during these workshop sessions. The professional caregivers did not serve as a proxy for the residents in this study but as key stakeholders in their own right, with opinions on providing care for people with dementia [47].

By bridging existing research insights with professional caregivers’ lived experiences, we established a shared understanding of opportunities for embedding ABT in residential dementia care to augment existing care activities, routines, and habits with everyday sounds. Furthermore, we discuss how these opportunities grounded in both practice and literature motivate further research on the implementation and evaluation of audio-based and experience-centered technologies in healthcare settings.

## 2. Materials and Methods

Professional caregivers working in residential care facilities for people with dementia generated opportunities for integrating ABT in practice in three workshops using a participatory approach [45,48]. We sensitized the professional caregivers on the potential benefits of ABT [49], explored the personal lived experiences of professional caregivers [50], and facilitated group discussions between these relevant stakeholders [51]. With this approach, we aimed to bridge insights from academic research to everyday practice and vice versa.

### 2.1. Vita: Audio-Based Design Intervention

**Figure 1:**
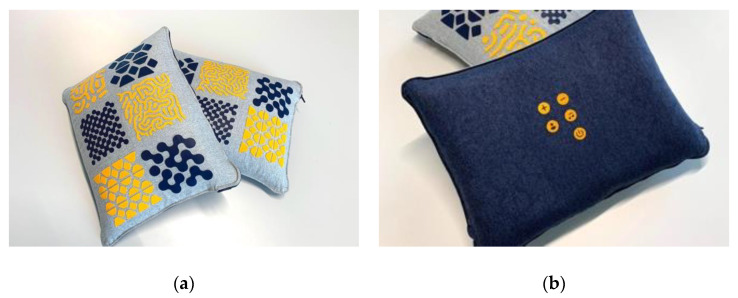
Vita served as an example of audio-based technology: (**a**) Vita is a cushion with conductive touchpads to play audio content and (**b**) with buttons on the reverse side to adjust the settings or volume.

The participatory workshops were conducted as part of a field study aimed at exploring the role of everyday sounds facilitated by ABT in a real-life care environment. This field study [22] was centered around the deployment of Vita, an audio-based design intervention (see Figure 1), in two residential care facilities for people with dementia. Vita is a cushion equipped with a speaker and six conductive sensors embedded in the cushions’ surface underneath colorful patterns of vinyl that serve as a safe and easy-to-use interface [52]. People with dementia can play audio files by touching one of six touchpads on the cushion. Upon touching such a touch pad, the corresponding sound starts to play until the resident stops touching it. This easily accessible interface enables people in advanced stages of dementia to play audio. Vita can be used in various ways in a care facility, such as at the table in the communal space, on the lap of the resident, or in bed in their private room. Vita was designed as a cushion so that it blends in with the interior of the care facility and is readily available for use. Caregivers and relatives can record, upload, and select personal audio content via an online platform. Vita is the outcome of a multidisciplinary collaboration between researchers, designers, and care practitioners and was developed in a codesign process involving people with dementia and their caregivers [53]. The participatory workshops served as introductory sessions for the professional caregivers overseeing Vita’s deployment in the care facility. The aim was to encourage and stimulate creativity in the use of Vita by professional caregivers during this field study by generating and designing concrete scenarios for using ABT in residential dementia care.

### 2.2. Workshop Procedure

At the start of the workshop, the participants were asked to introduce themselves to the researcher and each other, stating their name, profession (nurse or activity supervisor), care unit, and why they were interested in participating in the workshop. The workshop approach consisted of three consecutive steps to explore the different areas of expertise of the caregivers. First, the professional caregivers mapped everyday activities in residential dementia care onto a 24-h timeline. Next, we asked the professional caregivers to interpret statements taken from literature on sound and dementia and to link these to their practice and expertise. Lastly, the researcher introduced the professional caregivers to Vita: a cushion-like sound player as a concrete example of ABT within everyday care.

#### 2.2.1. Step 1: Mapping Everyday Activities in Care

**Figure 2:**
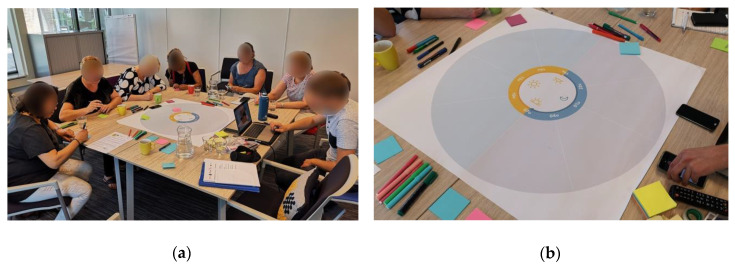
The caregivers mapped the typical day of a resident with dementia: (**a**) First, they were asked to reflect and make notes individually. (**b**) Next, participants were asked to share their thoughts with the group and to place their notes on the 24-h timeline.

The goal was to build an overview of the everyday care schedule and activities in residential dementia care [50]. During this step, the professional caregivers were asked to outline the typical day of a resident in a care facility (see Figure 2). A poster with a circle representing a 24-h timeline was provided to support the professional caregivers in mapping the daily activities to a specific timeframe and in sharing their views with the group [51]. The poster was placed in reach of all participants who were given pens, paper, and post-it notes to make individual notes or sketches.

First, the professional caregivers were asked to individually think about typical everyday activities in the care facility and to make personal notes. Giving professional caregivers an individual exercise stimulates participant engagement and generates more in-depth topics [45]. Next, each caregiver was asked in turn to read their notes aloud and to place it on the circular 24-h timeline. Participants were then given the opportunity to respond and discuss the notes in the group to stimulate discussion between the professional caregivers [50].

#### 2.2.2. Step 2: Relating Research to Everyday Care Activities

Step two was to inform the professional caregivers about academic research and evidence on how sound can support people with dementia and to broaden their understanding of how sound could contribute to their everyday practice [49]. They were provided with 12 statements collected from relevant literature on sound in general and more specific literature on sound in care facilities for people with dementia (see Table 1). To offer a clear overview of the research claims, we provided three categories of cards: nuisance (red), application (blue), and emotions and behavior (green). These cards guided and inspired the participants to design new scenarios for ABT [54].

**Figure 3:**
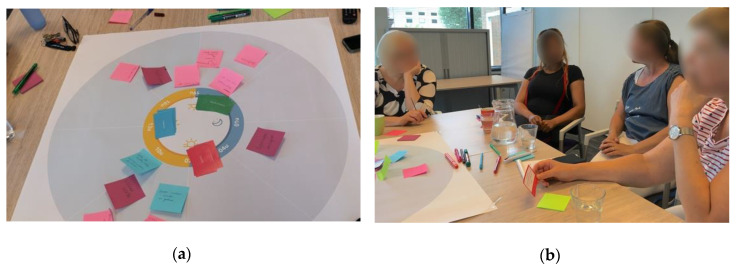
During the second step of the workshops, the professional caregivers were asked to map statements from literature to practice: (**a**) The cards with statements from literature were stacked in piles, face-down on the poster as in a board game. (**b**) Each caregiver was asked in-turn to draw a card and to relate the statement to their own experience, after which the group discussed the statement.

The cards were stacked according to category and placed face-down in the middle of the poster. Each caregiver was asked in turn to draw a card from one of the piles, to read the statement out loud in the group, and to formulate if they could relate this statement to their own experience (see Figure 3). The aim of these cards was to provide a playful way of presenting insights from academic research and to include all participants in the discussions [54]. Next, other participants were asked to react to the statement in order to stimulate a group discussion where professional caregivers could compare potential similarities and differences in the interpretation of the statement. After the discussion, the caregiver was asked to place the card on the 24-h timeline where the specific statement from literature was applicable. If the statement did not apply to a particular time or activity, professional caregivers could place the card in the center of the poster.

#### 2.2.3. Step 3: Designing Scenarios for Audio-Based Technology

**Figure 4:**
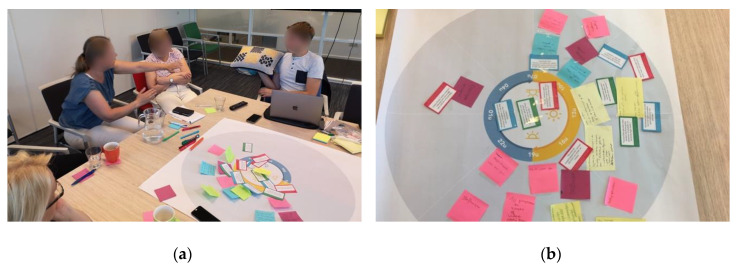
Lastly, the professional caregivers were asked to develop concrete use-cases for Vita: (**a**) The researcher explained the design rationale of Vita. (**b**) The professional caregivers were asked to think of specific cases to use Vita, which was then discussed during the final group discussion.

Step three bridged the insights from step 1 and step 2 to design potential scenarios for using ABT in dementia care (see Figure 4). Here, Vita was presented as a concrete example of an audio-based technology to cue the professional caregivers in generating ideas and concepts for adopting ABT in their practice. The primary researcher explained the design rationale of Vita and gave instructions on how the device works. Vita was passed between the professional caregivers to allow them to hold the device and to explore the interface. Next, the professional caregivers were asked to individually write down several concrete scenarios for using Vita on the large yellow post-it notes, as depicted in Figure 4. These scenarios were to include a specific resident, an activity, a timeframe, and potential audio content. Finally, the professional caregivers were asked to share their scenarios with the group and to place their post-it notes on the timeline to stimulate a final group discussion on the implementation of Vita and ABT in general, which concluded the session.

### 2.3. Participants and Setting

In total, 18 professional caregivers who work in residential dementia care participated in the workshops, divided over three sessions (as depicted in Table 2). Nine caregivers were part of the nursing staff responsible for everyday care of the residents, such as washing, dressing, or eating. The other nine were activity supervisors who organize and accommodate social daytime activities, such as music sessions or art therapy. All participants were female, except C18. The workshops took place at two care facilities in the Netherlands that both accommodate long-term residential care for people in advanced stages of dementia. We conducted one session at care facility A in Eindhoven with eight professional caregivers (C1–C8) and two sessions at facility B in Arnhem, with six (C9–C14) and four (C15–C18) professional caregivers, since not all professional caregivers at location B were able to attend the same session due to full agendas.

### 2.4. Data Analysis

The participatory workshops were audio-recorded and later transcribed verbatim. The primary researcher analyzed the transcriptions by conducting thematic analysis, following an inductive approach [57]. Statements relevant to the research question were clustered in codes and further refined into the themes discussed and iterated in consultation with the coauthors.

### 2.5. Ethics

The research activities in this paper were part of a field study on the deployment of Vita, which was approved by the Ethics Review Board of the Tilburg School of Social and Behavioral Sciences, Tilburg University (reference number: EC-2019.22) in which the codesign sessions were reported as introductory sessions for the professional caregivers participating in the field study. The professional caregivers were briefed about the study’s aim in general and the participatory workshops in specific. Next, informed consent was asked and collected according to the General Data Protection Regulation (GDPR), granting permission to record and analyze the audio of the participatory workshop sessions and to use photographic documentation in academic publications.

## 3. Results

Our results provide insight into the perspectives of professional caregivers in residential dementia care on the following:

1. the added value of ABT in the residential dementia care process as ABT can provide rest and relieve from stress during the transition between activities, can initiate social connection during everyday care tasks; and can relieve boredom by supporting daytime activities and situational social experiences in the care space and
2. how the successful integration of ABT in residential care requires a person-centered approach adapted to activities and habits specific to a care unit or healthcare team and tailored to the residents’ needs and preferences by exploring their individual responses.

### 3.1. The Added Value of ABT in the Care Process

The professional caregivers expressed their motivations for using ABT in the care facility by discussing areas for application and the potential added value for both the resident with dementia as well as the care staff.

#### 3.1.1. Relieve Moments of Stress and Boredom

Nearly all professional caregivers (C1–C4, C6, C7, C9–C11, and C13–C18) mentioned how they would use sound to calm residents and to provide relief from stress. During moments when residents were not at rest due to boredom, “recognizable relaxing sounds” (C1) could be used as a nonmedical alternative to address agitated behavior. This potential application of ABT providing relaxing sounds can alleviate part of the workload of professional caregivers who often struggle with finding effective ways or tools to calm distressed residents, as illustrated by C15: “At the end of the afternoon, in the evening, a resident often says: ‘I have to go home, my mother is very ill!’, and then, you have to try to calm her, for example, with piano music”. In current practice, several professional caregivers (C4, C13, and C15–C18) often play music in the background (e.g., the radio) to prevent residents from becoming stressed “because, in our group, they become very quiet when we put on music” (C17). Two professional caregivers (C9 and C17) suggested that audio messages of family members might calm and reassure residents worrying that their partner or family members have forgotten about them:

“Her son always comes to visit on the weekends, but at some point, she becomes very restless when he is not there yet. Maybe a message [from her son] saying “see you mom, see you later” can help here”.

(C9)

However, the successful use of ABT should also address the need for moments of silence and rest as professional caregivers (C11, C13–C15, and C17) made it clear that not all moments require sound or music: “why does music always have to be on?” (C15). At specific times during the day, silence is preferred to not distract the residents from essential activities, such as eating. For instance, C17 always switches off the TV in the evening when residents are having dinner “because, otherwise, residents will keep on singing and will not eat” (C17). In several living areas, the TV or radio is often turned on to provide some background sounds, but caregivers indicated how loud music or the sound of the TV often have an opposite effect and leads to a restless and hectic atmosphere (C10, C12, C13, and C16), as illustrated by C13:

“Because, sometimes [I] walk into a living area, and then I think, ‘why is the TV on now?’—sometimes just on a children’s channel—and then there is a lot of sound! But, no one is watching, and the [terrible] things that are on TV … (sighs)”

(C13)

Most professional caregivers (C1, C4, C7, and C9–C18) saw opportunities to use audio-based interventions during transitions between scheduled activities. For instance, before scheduled daytime activities, when residents become “agitated if the activity is not immediately happening” (C9), or directly afterward, when “all residents must return to the living area at the same time” (C7), because “then you have to keep them busy” (C4). Similarly, ABT could be used directly before or after care tasks to calm residents (C1, C4, C7, and C10–C18). In the morning, sound could provide “a little bit of excitement that might give them a peaceful moment to get out of bed” (C18) or could be provided “after breakfast to [help them] stay awake because, then, there is no activity” (C11). Professional caregivers (C1, C10, C11, C15, C16, and C18) saw potential in using Vita to improve sleeping patterns: “if someone is very restless before bed and you give [them] Vita with a recognizable relaxing sound, [then] it can also contribute [to relaxation]”. (C1). According to (C2, C15, C16, and C18), quiet and peaceful sounds could help with “sleeping through [the night] and falling asleep […] for people who wake up at night or [are] unable to fall asleep” (C16).

In summary, there is potential for ABT to support the care process in relieving moments of stress and boredom in the care space and in easing moments of transition to other activities or care tasks, but ABT should also offer room for silence and rest.

#### 3.1.2. Facilitating Playful and Engaging Daytime Activities

Professional caregivers suggested that ABT can support scheduled daytime activities that offer playful social experiences, such as music sessions or reminiscence therapy (C1, C3–C10, C13, C15, C16, and C18). Most professional caregivers (C1, C3–C9, C12, and C15–C18) had experience in providing sound-related activities during one-on-one sessions with the resident to specifically focus on multisensory stimulation of people in advanced stages of dementia. Professional caregivers (C1, C4, C5, C7, C9, C10, C12, C13, C15, and C18) also stated how sound could be a useful tool to support group activities involving multiple residents of the care facility, such as music activities (C1 and C7). Vita could facilitate an alternative group activity in the common living area for residents who do not attend daytime activities, for instance, during “periods between breakfast and lunch and, then, afternoon between lunch and dinner, when there are no activities and visitors” (C10).

However, it was also remarked that these group activities in the living area might not work (C2, C5, C12–C13, and C17), as it “is difficult in a group in the living room; if people do not want to listen, they can go read or something, but they will still hear it”. (C13). Daytime activities can produce unwanted sounds and become a nuisance for other residents and professional caregivers who are not partaking in those activities but still want and have to reside in the communal living area (C15 and C16). For instance, “when there is an activity in the hallway and the doors need to be open because people want to walk back and forth, […] that causes a lot of noise and disturbance”. (C15). Therefore, when using ABT in a real-life care space, the existing layer of ambient sounds and the acoustical impact of ABT need to be considered. Unwanted environmental sounds such as the dishwasher (C1, C2, C11, and C15), deliveries of meals or other supplies (C13, C14, and C16), and vacuum cleaners (C13 and C16) can cause restlessness among the residents or can disrupt daytime activities, as was the case for C13: “Someone started to vacuum the common space while I was holding an activity. You can notice it in the residents. They get restless”. During these moments, additional sounds or sound-related activities are often unwanted or not desirable as this might cause stress for the residents and the professional caregivers who already have to cope with heavy workloads and time constraints:

“Yes this is very personal, but I had quite a burnout myself, and when I had to come back to work for the first time, I was also very anxious because, over here, a resident was having a pedicure [and], over there, six people were wandering. The doors [to the hallway] were open. Someone had turned on the television because that is cozy, and there I was standing behind the counter as a frightened person thinking ‘what is going on here?’”

(C16)

Often, residents sleep during the day, but these daytime naps can mix up day-to-day structure and can disrupt sleeping patterns and sleep quality. For instance, “when there are no scheduled activities, she [a resident] is just staring ahead or taking a nap” (C10). Professional caregivers (C10, C11, C13, C16, and C18) want to use sound to animate residents during the day to prevent them from falling asleep and to support a daily wake–sleep structure. Most professional caregivers (C1, C2, C4, C6, C7, and C9–C18) indicated how ABT could be used in the care space to initiate spontaneous, playful activities by using pleasant and exciting sounds “to excite them a bit, so they do not disturb their rhythm of day and night, and they stay awake” (C13). From the experience of caregivers (C1, C2, C4, C7, and C15–C18), music effectively animates residents to make them more active or to wake them up in a spontaneous and meaningful way:

“I have someone who, uhm, when I always turn on the radio with jazz and blues, […] she immediately starts dancing then, and that reminds her of her past too”.

(C1)

Professional caregivers expressed the value of ABT to support daytime activities and to instantly initiate spontaneous activities that provide social engagement in the care space to provide structure and daily rhythm. Nevertheless, the deployment of such interventions and activities requires careful consideration of the existing acoustic environment and the impact of augmenting this environment with additional sounds.

#### 3.1.3. Support Social Interaction in the Care Process

The professional caregivers considered how ABT can offer cues for social interaction during their different care tasks within the daily care process. They (C1–C4, C6, C9, C10, and C12–C18) suggested a potential value of ABT to initiate and support social interactions during one-on-one activities with residents who are “in advanced stages of dementia—to make contact—as it is often difficult to make contact with them” (C14). Playing recognizable sounds and listening together can evoke broad topics of conversation (C2, C3, C10, C12, C13, C16, and C18). Caregivers suggest that sound could “evoke memories, so you also get people talking about it” (C2). In this context, professional caregivers (C1, C2, C6, C9, C12, C13, C15, C17, and C18) saw potential in using Vita to play familiar sounds to support reminiscence and to evoke emotions to facilitate meaningful and socially engaging experiences:

“That can be related to hobbies or something—or to work—or baby sounds if you happen to have worked in child care […] when you have one-on-one conversations about their work, hobbies, or past, then you also come across those sounds”.

(C15)

Professional caregivers (C2–C4, C6, and C13) made it clear that activity supervisors and the nursing staff have different perspectives and opinions on the use of ABT with residents. Residents can behave differently during organized daytime activities (e.g., music club) and when residing in the living area, as illustrated by (C2) (nurse): “No, she [activity supervisor] wants to use it with a specific resident, but we [nursing staff] have a different opinion on that. But, that is the difference between people who do nursing and colleagues [who supervise activities], whether residents are below [in the activity space] or whether they are above [in the living area]” (C2). Therefore, to understand how ABT can support different facets of everyday dementia care, it is required to incorporate the different functions and care tasks of the professional caregivers, such as providing activities, household tasks, or personal care. For instance, C15 supports residents in performing everyday routines such as getting up in the morning or assisting with bathing and uses music to reduce awkwardness and to make these tasks more enjoyable for her and for the residents:

“With physical care, which is quite stressful or difficult for several people and annoying, music can certainly help. […] I often sing songs with the people while providing morning care because, then, you can get a lot more done. I make a song from the activity or task that I am doing at that moment, especially with people in the advanced phases, so you can still try to make some contact. […] The morning care of one of the residents is becoming increasingly difficult for her and us. [We could] accompany it with soft and friendly piano music, because she is very interested in that, to start the day off right”.

(C15)

Listening to music was mentioned by C1, C4, and C15 as a useful way to connect with residents who have difficulty in initiating and engaging in a conversation: “especially when people are in advanced stages, you can still make contact that way” (C15). These moments of contact can also be with family members who visit the care facility and often struggle to hold a conversation or to make contact with their relative with dementia (C15 and C18). The professional caregivers had previously experienced the value of sound in supporting nonverbal contact with people in advanced stages of dementia and those who are unable to communicate verbally:

“Hearing is the last [sense] to disappear. […] You often see people whose dementia has progressed [being] involved in those activities [with sound] and they still respond by opening their eyes, but they can no longer speak. […] The hearing remains!”.

(C2)

ABT can offer opportunities to stimulate social connection during care activities, household tasks, or personal care by cueing conversations and by evoking nonverbal responses that reduce awkwardness and support difficult tasks in the care process.

### 3.2. Need for a Person-Centered and Reflexive Approach

The professional caregivers strongly expressed how the successful integration of ABT in everyday practice demands a person-centered approach that addresses the needs and situation of the residents with dementia and how reflexivity is required to explore and evaluate the in-context responses of the participants.

#### 3.2.1. Person-Centered Approach

Nearly all professional caregivers (C1–C7 and C9–C18) indicated that the use of ABT would strongly depend on the person with dementia as it “can certainly make a contribution, especially if they are open to it, but it is not for everyone” (C2). Sound content was seen as crucial in evoking meaning from residents by professional caregivers (C1, C5, and C9–C16). For instance, it is necessary to understand the preferences of the residents, and “you must first filter out what is pleasant because what is pleasant for me is not pleasant for you (C11)”. Professional caregivers (C2, C4, C7, and C10–C18) imagined themselves in the situation of the residents and stated how responses to sound apply “not only for people with dementia, [but] it also applies for yourself” (C14). Similarly, professional caregivers (C7 and C10–C18) linked discussions on sound in the care home to their personal life or household. Therefore, professional caregivers (C12 and C14–C16) suggested looking at the social and cultural background of the residents for meaningful audio content. For instance, C12 has a migration background and grew up in a context different from a typical Dutch household:

“Oh, I always put the radio on: when I get up, when I take a shower, sing[ing] along in the shower. Yes, I also think it depends on the culture too. There were always many people in my house, it was always busy, and I am not used to silence at all”.

(C12)

Similarly, professional caregivers are required to evaluate and monitor potential negative responses to sounds that evoke emotionally difficult memories and, as a result, to confront people with their dementia or to cause feelings of sadness. For instance, C11–C13 discussed how some residents became sad when listening to music or while reminiscing during daytime activities. However, feelings of sadness or nostalgia are not necessarily harmful as “those are also feelings to talk about” (C13). Furthermore, professional caregivers (C2, C3, C9, C11–C13, C16, and C17) expressed concern that listening to certain sounds can cause rest to one and unrest to the other: “you put on, for example, chirping birds because that is so soothing, but then one resident thinks: ‘those rotten birds, can’t they shut up?’” (C13). The professional caregivers discussed the complexity of using ABT in a communal space in a care facility where multiple people with different backgrounds have to live together:

C14: “Yes, sometimes, I just do not want any sounds at all. I do not have to have sound around me all day—or music”.

C12: “Oh, but I do, I prefer music all day long!”

C13: “Yes, but then, suppose that you two are sitting in one communal area room? That is the same with that television and radio [that is always on]!”

The workshop outcomes also revealed how professional caregivers adopt different routines and habits in their current practice in terms of using music during everyday care. For example, a discussion between two professional caregivers (C15 and C17) illustrated how different care units within the same care facility could have different opinions on using music during eating activities based on the different responses of the residents within the same care unit:

C16: “A world of difference between your home and ours, you cannot imagine it!”

C17: “Yes, with us, it just helps at breakfast to turn on music, to make them eat. That makes a huge difference!”

C15: “Yes, I find that so contradictory. That is not possible with us because, during dinner, you do not have music on, you sit at the table, eat with the people, [and] possibly talk, but you have no music in the background”.

The personal reflections and discussions of the professional caregivers revealed how a person-centered approach is required to involve residents in sound-related activities and that a one-size-fits-all approach is not desirable for people with dementia.

#### 3.2.2. Reflexivity and Continuous Assessment

The above examples illustrate the variety in the responses of the residents to sound. Even though professional caregivers have particular motivations for using ABT, as reported in the previous sections, there is no guarantee that these benefits and desired effects will apply for all residents. Professional caregivers (C2, C4, C5, and C14–C18) argued that the application of such interventions “depends on the resident’s condition” (C2) and can strongly vary over time:

“We try something with a resident, and today, it is going well and, tomorrow, it is not. The day after tomorrow, it works again, and after three weeks, it stops. There is nothing more variable than a person with dementia, and the group dynamics also change, depending on who of the caregivers is working. [There are] quite a lot of factors you cannot do much about”.

(C15)

Therefore, ABT should not necessarily be used at fixed points in time but should be used to support certain situations in care that can arise during the day. For instance, professional caregivers (C2–C4, C6, C9, C11, C13, and C15–C18) indicated that they would use sound to calm residents “in case of unrest, which also differs per person and can happen at any time, not just at a specific point in the day”. In that sense, professional caregivers (C1–C3, C6, C12–C16, and C18) expressed that using ABT to evoke responses is a matter of “trying it out” (C6) by assessing their responses and “feel[ing] for yourself if it is going well” (C16). In using Vita, professional caregivers are required to continuously assess the individual responses of the residents to explore the specific potential value for each resident:

“I am curious how the residents [will] react to the pillow when they are restless or when they are just relaxed and at rest, if they then react differently”.

(C3)

Reflexivity and continuous assessment are required in exploring individual responses to ABT, with room for experimentation for professional caregivers to question and investigate their interactions with the residents in context via introspection as they occur as well as to share their perspectives with colleagues.

## 4. Discussion

Our participatory approach elicited a range of views and opinions from dementia care professionals about the potential benefits of introducing and incorporating ABT in their everyday working environment to support the care delivery process. We contribute to existing literature by synthesizing our analysis of the results into an overview of novel opportunities to integrate ABT in residential dementia care. Furthermore, we discuss how care professionals are vital stakeholders in facilitating the in-context use of technology and how the implementation of ABT should address their specific lived experiences and needs, such as care tasks, working culture, and multidisciplinary sharing of expertise.

### 4.1. Novel Opportunities for Integrating ABT in Residential Dementia Care

Firstly, ABT can provide new opportunities to support the transitions between scheduled activities in daily dementia care by offering peacefulness, distraction, or alternative activities. These moments of transition are identified as hectic and cause restlessness in both staff and residents. The literature reports that sound can provide a sense of safety and structure and can influence mood positively in dementia care environments [25,27,28,31,40]. Professional caregivers shared this view that sound can influence mood and support day-to-day structure and, most importantly, identified moments between activities as an opportunity for ABT to facilitate the anticipation, transition between, and the closing of daytime activities by countering impatience or by relieving nervousness.

Secondly, professional caregivers view ABT as a solution to improve the daily care process by providing meaningful audio content and by establishing verbal or nonverbal communication to enhance social interactions. In addition, ABT could also accommodate intimate moments in personal care, such as bathing or dressing, to contribute to the experience of both the residents and the caregivers. Therefore, the significance of sound in shaping social relationships among residents and between residents and professional caregivers should be recognized. This builds further on research that demonstrates how professional caregivers can establish an equal form of communication during moments of care by listening to or by playing sounds together with residents [9,22], as sound allows people to connect as it propagates through the care space [23].

Thirdly, the caregivers expressed how situational or opportunistic use of ABT during everyday care can evoke immediate and pleasurable in-the-moment experiences in the everyday living environment. However, to evoke these meaningful responses, the professional caregivers concluded that a person-centered approach is needed to consider the personal and sociocultural background of the residents. Research shows that people with dementia can associate sound to emotionally valued past experiences to evoke meaningful conversations with their relatives or caregivers [19]. The use of sound and music in scheduled daytime activities has been well established in relevant literature [7,11,19,37]. Sound and, in particular, music is referred to as a *universal* language [58] and can facilitate communication and social interactions with residents with aphasia or who have difficulty communicating verbally [16,22]. By adopting a person-centered approach and by engaging in pleasurable experiences with the residents, the care staff can create a positive atmosphere that reduces emotional workloads and contributes to the wellbeing of both the residents and the staff in residential care [59]. Thus, ABT can offer meaningful audio content to support professional caregivers in including residents in enjoyable and accessible daytime activities by overcoming their challenges with engaging residents in a social setting.

Lastly, professional caregivers and people with dementia in residential dementia care require moments of silence and peacefulness during activities that are important for the health of the residents. The adoption of ABT in dementia care requires careful consideration of the existing sonic environment and the potential interference of additional sounds that might disturb residents or staff. For example, communal eating might be an activity where additional sounds negatively impact the shared experience [60]. Therefore, ABT should be designed in a way so they can also offer moments of silence in the care space to provide opportunities where residents and professional caregivers can unwind without experiencing unwanted auditory stimuli. This builds on research on acoustics in care facilities that stresses the need for regulated ambient noise levels in care environments to reduce stress and to prevent agitation among residents and staff [61]. With new forms of ABT, such as “sound zone technology”, individual listening experiences could be provided in communal areas to limit sound disturbances between individual listeners through a specific speaker setup [62]. We conclude that more research is required to gain a full understating of how ABT can cater universally and for the individual at the same time.

### 4.2. The Needs and Lived Experiences of Care Professionals

Our results illustrate how different care units within the same care facility could have different routines or processes in providing care or that residents in different living areas have contradictory attitudes towards ABT that provide music or sound. Professional caregivers from the nursing staff, e.g., providing care at night, had utterly different motivations and approaches towards using ABT compared to colleagues who only supervised daytime activities. The different care tasks that are performed by a team of care professionals might be often overlooked in the design of ABT and missed in academic research. We argue that the design and development of ABT needs to consider the lived experiences of the care professionals and the workplace culture as caregivers are an integral part of the system in mediating technology and in engaging people with dementia in social activities [22,63,64].

The professional caregivers stated that successful use of ABT in context requires a person-centered and reflexive approach. This approach corresponds with the reflexivity required in the professional thinking of caregivers working in healthcare environments in general [65]. This reflexive process is mostly applied in complex clinical situations and can heavily vary between different caregivers as they have different experiences and perceptions of the perceived problems [66]. Recent research highlights how professional caregivers have not only other approaches in care but also different views on making sense of the actions and expressions of people with dementia [63]. Therefore, the professional caregivers appreciated the participatory workshops as they have a need to exchange their individual perspectives and to learn from each other’s experiences in providing care.

The participatory workshops served as a co-learning process by creating a setting that encouraged the exchange of expertise and knowledge between the researcher and the professional caregivers and among the team of caregivers themselves. The care staff sharing their concerns and opinions with technology developers and care organizations is crucial to prevent resistance in implementing or adopting new technologies [67], as healthcare teams often have different levels of interest or are misinformed about the effort and potential benefits [68]. This collaborative aspect of working in dementia care shows potential for further consideration in ongoing research, as we stress the need for multidirectional and multidisciplinary sharing of insights and ways of working to develop a shared understanding in using technologies such as ABT in residential care. This continuous knowledge exchange with regard to utilization and advantages contributes to the broader acceptance and implementation of technologies such as ABT and encourages its use by the care staff in everyday care practice.

### 4.3. Grounded Motivation for Future Field Studies

Adoption and support in using technology by care professionals is an essential requirement for field studies that aim to evaluate or iterate newly designed technologies [42,69,70]. The opportunities presented in this paper are the outcome of reflections and discussions with and from professional caregivers that were cued and triggered by matching academic insights to their personal experiences and practical expertise of providing care. As a result, these insights relate heavily to the existing care structures and activities of the involved care organizations and may differ between care institutions, countries, and cultures. Therefore, we do not present these opportunities for ABT as rigid guidelines but rather as a practice-informed foundation for future research on the implementation and evaluation of ABT in real-life care settings. We aim to broaden the scope of future work on ABT in care environments to explore the values and perspectives of critical stakeholders designated to facilitate and to use these technologies in context. Hence, further in-context and longitudinal evaluation studies of ABT is essential to generate knowledge on the effectiveness and the successful implementation of meaningful sounds to augment care practice.

## 5. Conclusions

We have explored the integration of audio-based technologies in dementia care by consulting the expertise of 18 professional caregivers working in residential dementia care during three participatory workshops. The study outcome reveals opportunities for ABT to add value in the care process and insight into the needs and experiences of professional caregivers to enable the sustainable use and implementation of ABT in dementia care. We present these study outcomes as a well-motivated foundation grounded in practice to steer future research on the adoption and evaluation of technologies such as ABT in care settings. The workshops facilitated a multidirectional sharing of knowledge with literature as a starting point for professional caregivers to understand the possibilities of ABT and the theories behind it. The professional caregivers were able to build on their experiences of environments, care structures, responsibilities, and people to provide insights on operating ABT in practice. They were also able to extend the discussion and to consider the different perspectives and practices of colleagues. While there is a body of work that focusses on the impact and responses of people living with dementia to audio content, it is still unresearched how professional caregivers negotiate the daily activities to bring about change with audio-based technology. This study contributes to this gap in the literature by allowing caregivers to bridge theory and to practice and provide a well-motivated foundation grounded in practice for future research on the adoption and evaluation of audio-based and experienced-centered technologies in care settings.

## Figures and Tables

**Table 1:** The cards contained statements and conclusions of relevant academic research to inform the professional caregivers of the opportunities and challenges of audio-based technology (ABT) for dementia care.

Category	Statements from Literature	Ref.
Red cards: potential nuisance of sound in a care environment.	“If a person cannot give meaning to a sound, it is considered noise”.	[55]
	2. “Sound can make people with dementia distracted, nervous, or scared”.	[30]
	3. “You can close your eyes but not your ears”.	[29]
	4. “Unpleasant sounds can cause stress and irritation in a care home”.	[56]
Blue cards: application areas of ABT.	5. “Pleasant sounds can mask unwanted or annoying sounds”.	[30]
	6. “Sound can evoke social interactions between people with dementia and caregivers”.	[21]
	7. “Sounds can provide structure and routine in a care facility”.	[40]
	8. “Everyday sounds can evoke memories related to those sounds”.	[19]
Green cards: emotional and behavioral responses to sound.	9. “Pleasant noises can calm a person with dementia”.	[27]
	10. “Personal sounds can support identity and selfhood in dementia”.	[14]
	11. “Joyful sounds can stimulate and revive people”.	[24]
	12. “By listening to a pleasant sound, someone can feel at ease and safe”.	[28]

**Table 2:** In total, 18 professional caregivers participated in the participatory workshops, divided over three sessions organized at two different care facilities. The professional caregivers were either nursing staff (N) in charge of everyday care tasks or activity supervisors (A) who provide daytime activities. All caregivers were female (f), except C18 (m).

Care Facility A								Care Facility B									
Session 1								Session 2						Session 3			
C1	C2	C3	C4	C5	C6	C7	C8	C9	C10	C11	C12	C13	C14	C15	C16	C17	C18
A	N	A	N	A	A	A	A	N	N	N	N	A	A	N	N	N	A
f	f	f	f	f	f	f	f	f	f	f	f	f	f	f	f	f	m
